# Technical and clinical validation of the Allergen BioCube^®^ for timothy grass

**DOI:** 10.1002/iid3.143

**Published:** 2017-02-02

**Authors:** Endri Angjeli, Paul Gomes, Keith J. Lane, Linda Stein, Mark B. Abelson

**Affiliations:** ^1^ Ora, Inc.AndoverMassachusettsUSA; ^2^ Department of OphthalmologyHarvard Medical SchoolCambridgeMassachusettsUSA

**Keywords:** Allergen BioCube^®^, allergy validation, timothy grass

## Abstract

**Introduction:**

Field studies for allergic rhinitis (AR) commonly have inconsistent allergen concentrations and subject exposure patterns due to varying environmental conditions and subject behaviors. A technical and clinical validation study was conducted for the Allergen BioCube^®^ using timothy grass to confirm uniform allergen concentration and clinically relevant subject symptom responses.

**Methods:**

Allergen concentrations were verified by laser particle counts. Subjects (*N* = 14) with positive skin test reactions and no symptoms at screening received four 3‐h timothy grass exposures in the BioCube over consecutive days. Subjects evaluated nasal itching, sneezing, rhinorrhea, and nasal congestion while in the BioCube; Total Nasal Symptom Score (TNSS) was computed. Peak Nasal Inspiratory Flow (PNIF), Peak Expiratory Flow Rate (PEFR), sIgE blood tests, and Nasal Inflammation Score (NIS) were assessed. A correlation analysis was conducted for mean sIgE, skin test, and TNSS.

**Results:**

Uniform timothy grass concentrations were achieved in the BioCube, both spatially and temporally, at all subject positions. Mean TNSS increased substantially from pre‐exposure levels (0.36 ± 0.74 to 1.86 ± 2.14) to maximums of 7.07 ± 2.76 at 1.5 h and 6.71 ± 2.70 at 3 h BioCube exposure. Twelve (86%) subjects had TNSS increases ≥6 units. PNIF decreased 12–24% from baseline at 3‐h BioCube exposure. NIS increased (baseline = 0) to 3.7 (maximum score = 4). A low/moderate correlation (*r* = 0.485) occurred between mean sIgE blood levels and mean skin tests; neither sIgE or skin tests correlated with mean TNSS. However, subjects with high skin test scores or positive blood IgE tended to also have higher TNSS.

**Conclusions:**

The Allergen BioCube achieved technical and clinical validation for uniform timothy grass concentration and clinically meaningful AR sign and symptom responses. The Allergen BioCube can be used to assess the efficacy of therapies for reduction of AR signs and symptoms resulting from grass exposure.

## Introduction

Achieving consistent subject exposure conditions can be challenging in allergic rhinitis (AR) field studies. Allergen concentrations are highly variable in the environment, both between sites and at single locations within an allergy season and in different years. In addition, subjects may be exposed to multiple allergens simultaneously, making it difficult to assess which allergen is triggering a response. Exposure may also differ for subjects depending on how much time they spend indoors versus outdoors.

Environmental exposure units (EEUs, also known as allergy, exposure, or challenge chambers) control the variability in allergen concentration and subject exposure commonly associated with field studies by monitoring and adjusting conditions such as temperature, humidity, and allergen/air flow distribution 1, 2, 3. AR studies have been conducted in EEUs for ragweed, dust mites, grasses, trees, and cat dander 4, 5, 6, 7. Several EEUs have been validated for consistent allergen concentration and induction of AR signs and symptoms 8, and drug efficacy has been evaluated in EEUs 4, 9. The Allergen BioCube^®^ (Ora, Inc., Andover, Massachusetts) is an EEU developed to provide precise, consistent, and safe delivery of clinically relevant allergen concentrations. A technical and clinical validation study of the Allergen BioCube was conducted for timothy grass allergen 10. Timothy grass is native to Europe and is widely available in the U.S. as horse feed and lawn grass. The Allergen BioCube has also been validated for ragweed 11 and dust mites 7, used in drug efficacy studies 9, and shown to induce AR symptomatology similar to the natural environment 12.

While subject evaluations of their allergy symptoms are commonly used in AR studies, it is beneficial to also use more objective biomarker measures to corroborate subject symptom assessments. Yet some objective measures, such as blood IgE and skin tests, have shown variable correlation with clinical AR symptoms 6, 13, 14, 15. This article discusses the validation of the Allergen BioCube, using both subjective and objective sign and symptom measures.

## Methods

### Technical validation

**Figure 1:**
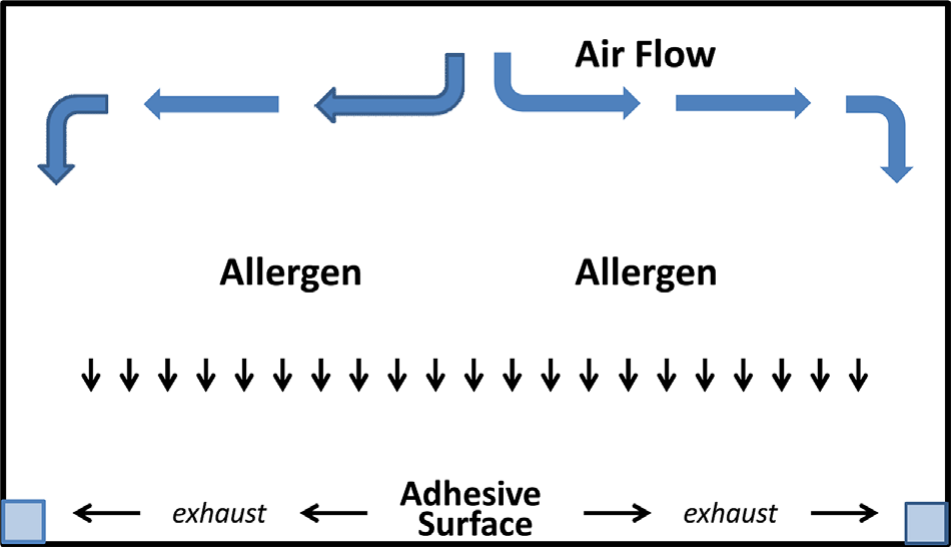
Air and allergen flow in the Allergen BioCube.

The Allergen BioCube is a 122 m^2^, Level 3 clean room environment that can accommodate up to 25 subjects and three qualified clinical staff. The distribution system transports the allergen at a pre‐determined rate and volume and mixes it with HEPA‐filtered air. The allergen/air mixture is radially distributed into the top of the room using laminar flow. Air is recaptured by return ducts that are flush with the floor along the perimeter of the room, HEPA‐filtered, and returned for recirculation (Fig. 1). An allergen buffer system at the BioCube entrance creates pressure to maintain air flow dynamics when a subject enters or leaves the room and serves as a cleaning (decontamination) unit.

#### Allergen concentration specification

For this study, timothy grass *(Phleum pratense)* (Greer Laboratories, Lenoir, North Carolina) was used in the Allergen BioCube, with purity certificates of analysis that indicated there was no fungi, bacteria, impurities, or any other biological matter in the grass sample. The specification for timothy grass concentration was 3000 ± 500 grains/m^3^.

#### Technical validation methods

Timothy grass concentrations in the Allergen BioCube were assessed during three 3‐h periods. Temperature was maintained at 20° to 26°C, and relative humidity was maintained at 35–55%. Once timothy grass distribution was initiated and air flow reached equilibrium, aerosolized grass counts were used to verify the concentration and maintain subject safety, ensuring that subjects would not receive exposure to higher doses of pollen than specified. Allergen concentrations were measured not only over time, but also at each subject position. Allergen levels were verified by Rotorod collection and laser particle counts, with equipment placed at the height of participants’ heads during validation testing to certify real‐time particle counts.

### Clinical validation

#### Clinical study design and methods

Subjects (*N* = 14) were evaluated at five study visits for 11 (+5) days outside of the grass allergy season. After screening and qualification (Day 1—Visit 1), subjects were exposed to timothy grass in the BioCube for 3 h per day on Days 8–11 (Visits 2–5, respectively). All subjects signed informed consent forms, and the study protocol and informed consent were approved by­­­ an IRB.

Key inclusion criteria were age (18–65 years), allergic rhinitis to a grass pollen documented by a self‐reported history of nasal symptoms for the last 2 consecutive years and a positive skin test reaction (wheal ≥ 5 mm larger than negative control) to a grass mixture and/or timothy grass within the past 24 months, and a history of moderate to severe AR requiring use of anti‐allergy medication in the last 2 years during allergy season. Exclusion criteria included having any significant illness that could interfere with the subject's health or the study parameters and/or put the subject at any unnecessary risk; a history of mild persistent, moderate or severe asthma within the preceding 5 years; compromised lung function at Visit 1 (<80% of predicted average PEFR); upper respiratory tract or sinus infection within the previous 2 weeks; current diagnosis of nasal polyps, severe septal deviation, nasolacrimal drainage system malfunction or chronic sinusitis; and history of anaphylaxis or poor tolerability of previously administered allergen.

Washout periods and no use of disallowed medications were confirmed prior to initiating study procedures. Disallowed medications included intranasal or systemic decongestants, leukotriene modifiers, and H_2_‐blockers (for 24 h); mast cell stabilizers (5 days); antihistamines, corticosteroids (inhaled, ocular, or intranasal), or topical skin medications on forearms (7 days); oral and parenteral corticosteroids, oxymetazoline hydrochloride, or ACE inhibitors (14 days); or other anti‐inflammatory, anti‐allergy, and other medications which in the opinion of the Investigator might have interfered with the study objectives, considered on a case‐by‐case basis.

#### Clinical validation methods

##### TNSS

Subjects’ total nasal symptom scores (TNSS, 0–12 unit scale) were computed as the sum of four individual symptom scores evaluated by subjects (nasal itching, sneezing, rhinorrhea, and nasal congestion, 0–3 scale, with 0 = none, 1 = mild, 2 = moderate, and 3 = severe). The individual scores were assessed pre‐BioCube exposure and every 30 min (±10 min) during the 3‐h timothy grass exposure in the BioCube, for a total of seven measurements per subject. TNSS in the BioCube reflected instantaneous TNSS score (iTNSS), that is, nasal symptoms at the time of evaluation. TNSS was recorded at Visits 2–5. A subject's overall TNSS was based on the subject's average TNSS over the 90‐ to 180‐min time period during BioCube timothy grass exposure, which represented a plateau of symptoms.

##### PNIF

Peak Nasal Inspiratory Flow (PNIF) was measured at Visits 2–5, pre‐BioCube exposure and every 60 min (±15 min) during timothy grass exposure in the Allergen BioCube.

##### NIS

Nasal endoscopy was conducted to grade nasal inflammation. The Ora Calibra™ Nasal Inflammation Score (NIS), a proprietary measure of nasal mucosal tissue inflammation and turbinate edema (Ora, Inc.), was used (0–4 unit scale, with 0 = No change from baseline imaging and 4 = no nasal passage opening for air to pass). The scale has been validated and was used in a blinded manner by grading photographic images. NIS was measured by the Investigator at the end of Visits 2–5.

##### Skin prick test

To participate in the study, a subject had to have a positive skin prick test to timothy grass and/or a grass mixture within the past 24 months. For subjects without such documentation, an allergen skin prick test was performed comparing the difference in wheal size for each subject's reaction to the grass compared to a placebo (saline). A positive skin test was defined as a reaction that was ≥5 mm larger than the negative control.

##### IgE blood test

A blood test for serum IgE (sIgE) was conducted for each subject. Up to ∼5 mL of blood was taken from each subject to determine allergen‐specific sIgE concentrations (antibody levels) to timothy grass and/or the grass mixture. The sIgE detection limit was 0.35 kUA/L, with a result >0.35 kUA/L defined as a positive sIgE blood test.

##### Safety measures

Safety measures included adverse events reporting, nasal exam, and peak expiratory flow rate (PEFR). PEFR was measured every 60 min (±15 min) during timothy grass exposure in the BioCube.

## Results

### Technical validation results

**Figure 2:**
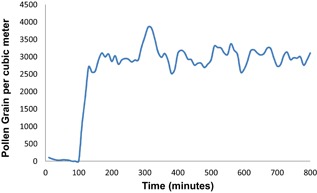
Timothy grass concentration in the BioCube was within the specification of 3000 ± 500^m‐3^ throughout each 3‐h testing period; levels of 2987 ± 42 ^m‐3^ were confirmed by laser particle counts.

**Figure 3:**
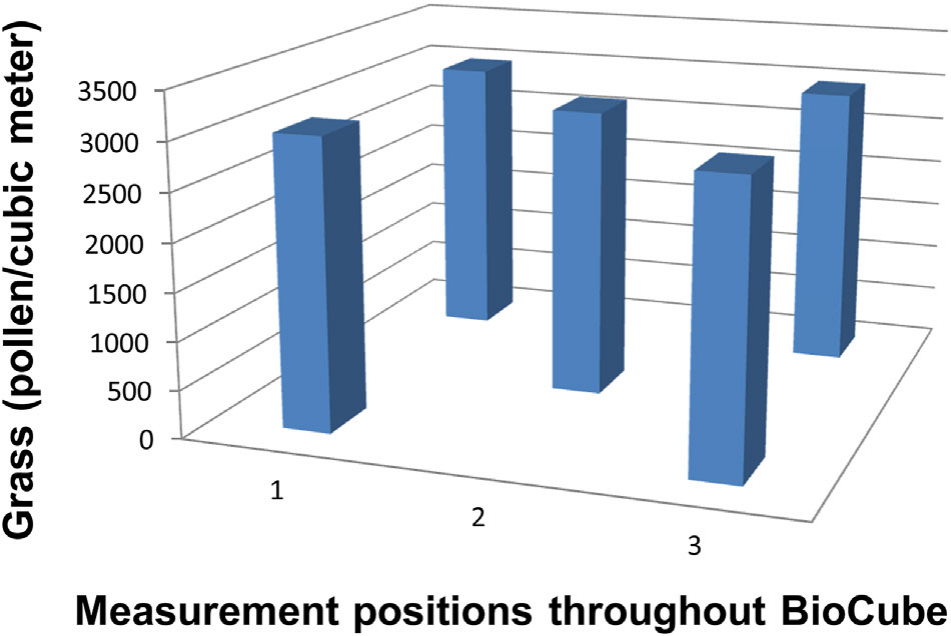
Timothy grass concentration was uniform, both spatially and temporally, at all subject positions within the BioCube, as confirmed by continuous laser particle count measurements.

The specification for timothy grass concentration of 3000 ± 500 grains/m^3^ was met throughout each of the 3‐h testing periods within the BioCube, as confirmed by laser particle counts of 2987 ± 424 grains/m^3^ throughout the study, as shown in Figure 2. Timothy grass concentration was uniform, both spatially and temporally, at all subject positions within the BioCube, as shown in Figure 3, as confirmed by continuous laser particle measurements at subject locations. Thus, the Allergen BioCube achieved technical validation for timothy grass.

### Clinical validation results

#### TNSS

**Figure 4:**
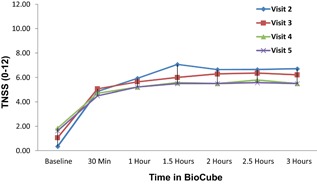
Mean TNSS increased after 3 h of BioCube ragweed exposure to 6.71 (±2.70) at Visit 2, and to 5.50 (±3.48) to 6.21 (±3.40) at Visits 3–5. Peak TNSS was 7.07 (±2.76) at 1.5 h at Visit 2.

Mean TNSS results (*N* = 14) during the Allergen BioCube timothy grass exposure sessions indicated clinically meaningful symptom responses, with observed mean TNSS scores increasing substantially from pre‐exposure levels at all visits. Peak mean TNSS of 7.07 (±2.76) occurred at 1.5 h at Visit 2; at 3 h, the maximum mean TNSS was 6.71 (±2.70), also at Visit 2 (Table 1 and Fig. 4). Mean TNNS was generally similar at all four BioCube sessions. TNSS scores at BioCube exposure sessions 4 and 5 were very similar throughout the study.

**Table 1:** Mean TNSS, Allergen BioCube timothy grass exposure

TNSS^1^ (*N* = 14), Mean (±SD) Allergen BioCube timothy grass exposure		
Pre‐exposure	1.5 h	3 h
Visit 2 (Day 8)		
0.36 (±0.74)	7.07 (±2.76)	6.71 (±2.70)
Visit 3 (Day 9)		
1.07 (±1.64)	6.00 (±3.01)	6.21 (±3.40)
Visit 4 (Day 10)		
1.86 (±2.14)	5.57 (±3.16)	5.50 (±3.48)
Visit 5 (Day 11)		
1.64 (±2.37)	5.50 (±3.46)	5.50 (±3.46)

^1^ Subjects assessed four individual nasal symptom scores (nasal itching, sneezing, rhinorrhea, and nasal congestion) on a 0–3 scale (0 = none, 1 = mild, 2 = moderate, and 3 = severe). The composite TNSS score (0–12 scale) was calculated as a sum of the individual symptom scores.

#### Individual nasal symptom scores

All four individual symptom scores also increased substantially from baseline during BioCube grass exposure, validating the BioCube for assessment of each of these symptoms (Table 2).

**Table 2:** Mean individual symptom scores, Allergen BioCube timothy grass exposure

	Allergen BioCube timothy grass exposure (*N* = 14), Mean (± SD^1^, ^2^)				
	Baseline^3^	Visit 2 (Day 8)	Visit 3 (Day 9)	Visit 4 (Day 10)	Visit 5 (Day 11)
Nasal congestion	0.20 (±0.41)	2.2 (±0.73)	1.86 (±0.94)	1.70 (±0.96)	1.78 (±1.01)
Nasal itching	0.00 (±0.00)	1.58 (±0.85)	1.38 (±0.71)	1.27 (±0.78)	1.27 (±0.94)
Rhinorrhea	0.07 (±0.26)	1.62 (±0.77)	1.41 (±0.97)	1.27 (±0.97)	1.25 (±1.07)
Sneezing	0.07 (±0.26)	1.27 (±0.91)	1.19 (±0.96)	1.00 (±0.89)	1.00 (±0.91)

^1^ Subjects assessed the four individual nasal symptom scores (nasal itching, sneezing, rhinorrhea, and nasal congestion) on a 0–3 scale (0 = none, 1 = mild, 2 = moderate, and 3 = severe).

^2^ Visits 2 through 5 reflect average of 1.5–3 h time points, reflecting a plateau of symptom responses.

^3^ Baseline was pre‐Allergen BioCube timothy grass exposure at Visit 2.

#### Subject TNSS scores

For each individual subject, TNSS increased from baseline, with an increase of ≥6 units in 12 (86%) subjects (10 [71%] subjects had increases of ≥7 units). The other two subjects had TNSS increases from baseline of five and four units. A maximum TNSS of ≥6 was reported by 12 (86%) subjects, and six subjects (43%) had TNSS of ≥10. The highest maximum TNSS score of 12 occurred in two subjects at Visits 3–5 (baseline TNSS of seven and two). One subject had a maximum TNSS of 11 at Visit 3 (baseline TNSS = 0), and three subjects had maximum TNSS scores of 10 at Visit 5 (baseline TNNS = 0 for two subjects and TNSS = 1 in the third subject).

#### PNIF

**Figure 5:**
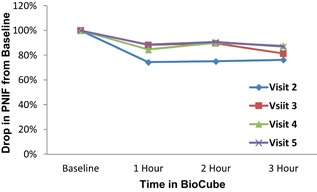
PNIF decreased up to 24% from baseline after 3 h of timothy grass exposure in the Allergen BioCube at Visit 2. PNIF percent decrease ranged from 12% (Visit 4) to 24% (Visit 2).

PNIF decreased up to 24% from baseline after 3 h of BioCube timothy grass exposure, supporting the onset of allergic symptoms in the BioCube with a more objective measure. Percent decrease in PNIF ranged from 12% (Visit 4) to 24% (Visit 2) (see Fig. 5).

#### NIS

NIS increased from a baseline of 0 to a score of 3.7 ± 1.6 (with a maximum possible score of four) by Visit 5, indicating increased nasal inflammation after timothy grass exposure in the Allergen BioCube.

#### Skin test

Thirteen of the 14 subjects had positive skin test reactions to timothy grass (i.e., a reaction that was ≥5 mm larger than the negative control). One subject tested positive for a grass mixture but not to timothy grass alone. Also see the *Discussion* section.

#### Blood IgE

Seven of the 14 subjects had positive blood sIgE values, and seven subjects had no detectable blood sIgE levels. Also see the *Discussion* section.

### Safety

Two adverse events occurred during the study. One subject had a sinus headache, and another subject had epistaxis. No reductions in PEFR>15% occurred, which would have resulted in discontinuation of a subject from the study. Nasal exams revealed no clinically significant findings.

## Discussion

This study provided technical and clinical validation of the Allergen BioCube for uniform timothy grass concentration and AR sign and symptom responses in timothy grass‐sensitive subjects. Timothy grass concentrations in the BioCube were consistent, both temporally and spatially, at all subject positions and were always within specification limits. Mean TNSS, individual nasal symptom scores, and PNIF and NIS results indicated a clinical response to timothy grass allergen in subjects at all four BioCube exposure sessions, with similar results at all sessions. The high level of technical and clinical precision achieved by the Allergen BioCube provided clinically relevant subject responses with a small number of subjects (*N* = 14); such precision can decrease potential non‐responders that might result from an inefficient exposure system.

It is important to assess individual nasal symptom responses in addition to TNSS; clinically, some patients may suffer more from one symptom than another, and some drug treatments for AR do not adequately treat certain symptoms (e.g., nasal congestion).

While mean TNSS responses were somewhat lower at Visits 4 and 5 than at Visits 2 and 3, the general trend was repeated at these later visits and was typical of EEU exposures 16, 17: a sharp initial increase in allergy symptoms, with continued but less steep increases throughout, or a leveling off or slight decrease towards the end of the 3‐h Biocube allergen exposure at each study visit. It is interesting to note that in the BioCube study, some of the highest individual subject TNNS occurred at later BioCube grass exposure sessions (Visits 3 through 5).

Priming did not occur in this study and was not needed to produce clinically meaningful sign and symptom responses. The lack of priming may have occurred because study subjects might have recently been exposed to other allergens, that is, perennial indoor allergens such as dust mites, or outdoor tree allergens (the study was conducted during tree allergy season but not grass allergy season in the Northeast). The role that priming plays, if any, in clinical reactivity and the mechanism by which priming might occur is not well understood.

Mean TNSS generally followed an overall pattern of escalation of baseline at each subsequent study visit, indicating a prolonged residual response to BioCube allergen exposure (with the exception that the average baseline mean TNSS score at Visit 4 was slightly higher [TNSS = 1.86 ± 2.14] than the Visit 5 mean baseline score [TNSS = 1.64 ± 2.37]). Results for individual subjects indicated this escalation of baseline pattern occurred for seven of the 14 subjects.

Other researchers have addressed the issue of correlation (or lack thereof) between skin tests, sIgE tests, and clinical reactivity 18. Huss‐Marp et al. conducted correlation tests for these parameters for 104 subjects with allergic rhinoconjunctivitis to grass pollen 13. That environmental study found that neither sIgE nor skin test results correlated well with clinical reactivity (assessed by direct nasal spray), while sIgE results highly correlated with skin test results. Lack of correlation between skin test results and clinical symptoms also occurred in a ragweed study of 31 subjects that included allergy testing in an EEU out‐of‐season as well as testing in a field study during the natural allergy season 6. In a dust mite study, sIgE levels correlated only somewhat with skin tests (13 of 35 subjects had both positive sIgE and positive skin tests for dust mites); subjects with positive sIgE to dust mites had greater clinical symptoms. An interesting finding was that positive skin tests for a different allergen (i.e., tree pollen) were correlated with clinical responses in the EEU, indicating a cross‐reactivity in subjects 19.

Correlation analysis for the total study population in the Allergen BioCube timothy grass validation study indicated a low/moderate correlation (*r* = 0.485) between mean sIgE and skin prick test (SPT) results, no correlation (*r* = 0.116) between mean sIgE blood levels and mean clinical TNSS responses, and no correlation (*r* = 0.192) between skin tests and TNSS. However, review of individual subject results showed a somewhat different story. Half (7) of the subjects had all three of the following characteristics: mean TNSS ≥ 6, positive SPT (≥5 mm), and a positive sIgE blood test. Eleven subjects had TNSS ≥ 6 and a positive SPT. Seven subjects had positive sIgE tests and TNSS ≥ 6. Seven subjects had positive SPT and positive sIgE blood tests.

**Figure 6:**
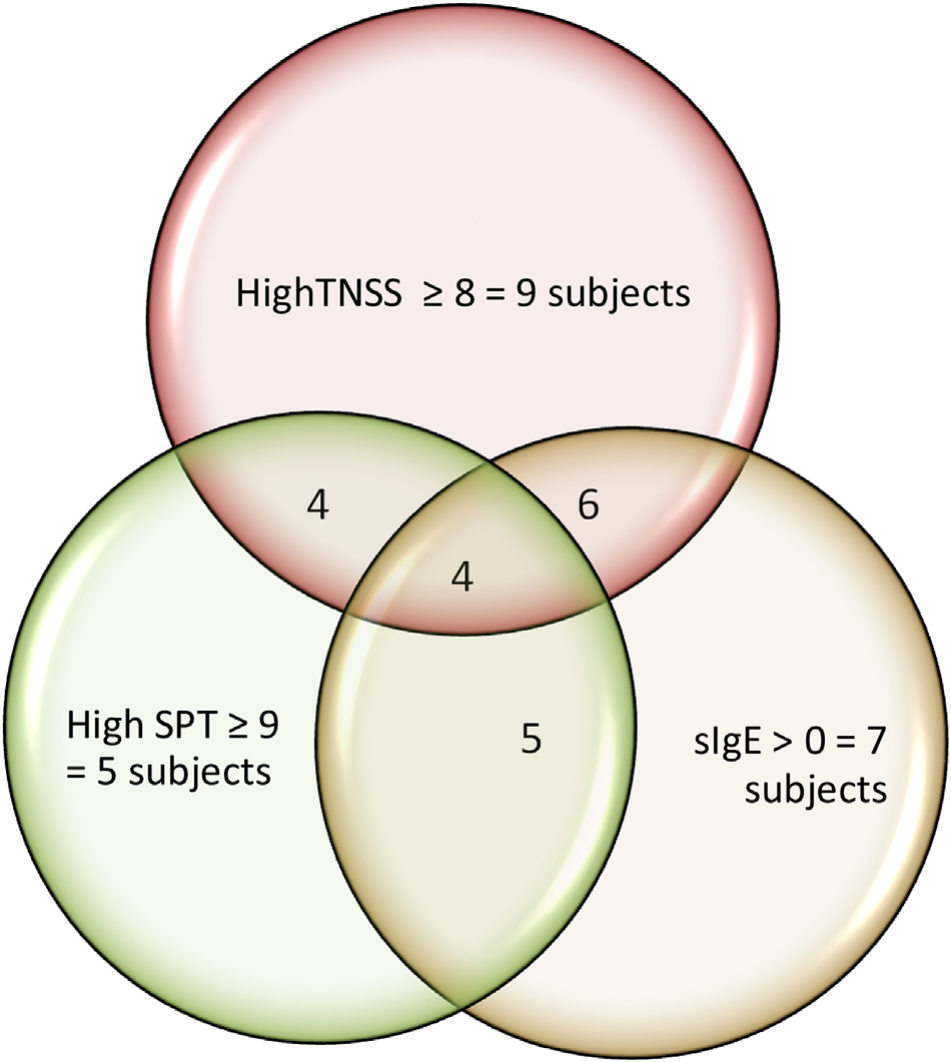
Subjects with high SPT (9–15 mm wheal) or positive sIgE tended to have high maximum TNSS (TNSS = 8–12). Four of five subjects with high SPT had high TNSS, and six of seven subjects with positive sIgE had high TNSS. All five subjects with high SPT had positive sIgE.

In addition, there was a tendency for individual subjects with highly positive SPT or positive sIgE blood tests to have higher TNSS. As shown in Figure 6, four of five subjects with high positive SPT had high maximum TNSS; six of seven subjects with positive sIgE blood tests had high maximum TNSS; and all five subjects with high positive SPT had positive sIgE. This tendency is similar to a 2‐day grass exposure study in another EEU (*N* = 22), in which strongly positive skin and blood IgE test responses to grass were also predictors of high nasal symptom scores from timothy grass exposure 17.

The clinical results of the three timothy grass studies discussed above support the use of EEUs for grass pollen testing. The validation of the Allergen BioCube and other EEUs contributes to their potential acceptance by regulatory agencies for expanded use in AR studies.

## Conflict of Interest

Co‐authors Angjeli, Gomes, Lane, and Stein are employees of Ora, Inc. Co‐author Dr. Mark Abelson, MD, is Founder and Chief Scientific Officer of Ora, Inc.
